# Dietary carbohydrate rather than protein intake drives colonic microbial fermentation during weight loss

**DOI:** 10.1007/s00394-018-1629-x

**Published:** 2018-02-20

**Authors:** S. W. Gratz, S. Hazim, A. J. Richardson, L. Scobbie, A. M. Johnstone, C. Fyfe, G. Holtrop, G. E. Lobley, W. R. Russell

**Affiliations:** 1 Rowett Institute, University of Aberdeen, Foresterhill, Aberdeen, AB25 2ZD UK; 2 Biomathematics and Statistics Scotland, Aberdeen, UK

**Keywords:** Faecal metabolome, Ferulic acid, Butyrate, Fermentation, Amino acids, Dietary fibre

## Abstract

**Purpose:**

High protein weight loss diets are effective in aiding body weight management. However, high protein and low carbohydrate intakes can alter colonic fermentation profiles in humans and may impact on colonic health. This study aims to identify the most important dietary contributors to colonic fermentation during diet-controlled weight loss.

**Methods:**

Overweight or obese male volunteers (*n* = 18) consumed a body weight maintenance diet (fed at 1.5× basic metabolic rate, BMR) followed by three weight loss diets (fed at 1× BMR) for 10 days each in a cross-over design. Weight loss diets were designed as normal protein (NPWL, 15% of energy from protein, 55% from carbohydrate), normal protein enriched with free amino acids and moderate amounts of carbohydrate (NPAAWL, 15% of energy from protein, 15% from free AA, 40% from carbohydrate) or high protein containing moderate amounts of carbohydrate (HPWL, 30% of energy from protein, 40% from carbohydrate). Faecal samples collected at the end of each diet period were profiled for dietary metabolites using LC–MS/MS.

**Results:**

This study shows that the NPWL diet only induced very minor changes in the faecal metabolome, whereas NPAAWL and HPWL diets decreased carbohydrate-related metabolites (butyrate, ferulic acid) and increased protein-related metabolites. Most faecal metabolites were correlated with dietary carbohydrate and not protein intake.

**Conclusion:**

This study demonstrates that dietary carbohydrate is the main driver of colonic fermentation in humans and that a balance between dietary carbohydrate and protein should be maintained when designing safe, effective and healthy weight loss diets.

**Electronic supplementary material:**

The online version of this article (10.1007/s00394-018-1629-x) contains supplementary material, which is available to authorised users.

## Introduction

The balance of macronutrients in the human diet has long been recognised as being centrally important for maintaining optimal health. Dietary carbohydrate intake forms the basis of our energy supply, whereas dietary protein provides the body with amino acids to maintain continuous protein turnover. Nonetheless, in settings of overconsumption, dietary carbohydrate and especially added sugars and easily digestible polysaccharides have come under scrutiny in terms of easily accessible energy [1]. In contrast, non-digestible forms of carbohydrate including resistant starches and non-starch polysaccharides are recognised as beneficial to human health via serving as substrates for fermentation by the microbiota resident in the human large intestine [2]. Health-promoting effects of dietary fibre (recommendation is currently at 30 g/day, [1]) are linked to the release of beneficial fermentation products such as the short chain fatty acids (SCFA), propionate, acetate and butyrate. Butyrate acts as an energy source for colonic epithelium and is actively involved in maintaining normal colonocyte turnover and possesses anti-carcinogenic and anti-inflammatory potential [3]. Microbial fibre fermentation also releases numerous phenolic compounds into the colonic environment many of which possess anti-oxidant and anti-inflammatory properties [4, 5]. Amongst these, ferulic acid as well as ferulic acid dimers and other metabolites are released in the colon following wheat-bran-enriched diets and are also absorbed into systemic circulation [6].

The role of dietary protein in colonic fermentation is less well understood. Older literature suggests that approximately 10% of dietary protein escapes digestion in the small intestine and acts as substrate for microbial breakdown into amino acids and possible fermentation to short chain fatty acids, branched chain fatty acids and ammonia in the human large intestine (for reviews see [7, 8]). Furthermore, colonic microbial fermentation of aromatic amino acids results in phenylacetic acid, 4-hydroxyphenylacetic acid and indole-3-acetic acid and additional catabolites [9]. Diets high in protein from red meat have been shown to promote colonic tumour development and endogenous formation of carcinogenic *N*-nitroso compounds [10–12], which led to the recent classifications of processed and red meat as human carcinogen and probable human carcinogen, respectively, by the International Agency for the Research on Cancer [13]. Nevertheless, high protein diets are very successful in aiding appetite control during weight loss in obese subjects [14, 15]. Consuming such weight loss diets with increased protein intake (mixed from meat, dairy and plant sources) and very low carbohydrate intake (including low dietary fibre) led to increased faecal protein fermentation products (including branched chain fatty acids, phenylacetic acid) as well as decreased fibre fermentation products (butyrate, ferulic acid) [16, 17]. It is not yet clear if increased protein and meat intake alone or in combination with decreased carbohydrate intake drives these shifts in colonic fermentation.

This study therefore aims to disentangle the potential effects of weight loss, dietary protein/meat and dietary carbohydrate on altering colonic fermentation by comparing three weight loss diets with varying protein and carbohydrate intakes. We designed diets to be body weight maintenance (M), normal protein weight loss (NPWL), normal protein weight loss enriched with free amino acids and containing moderate amounts of carbohydrate (NPAAWL) and high protein weight loss containing moderate amounts of carbohydrate (HPWL). We collected faecal samples at the end of each diet period and profiled the faecal metabolome.

## Subjects and methods

### Subject characteristics

Overweight or obese healthy, male subjects with a BMI > 27 kg/m^2^, stable body weight (< 2 kg change in the past 3 months) and fasted blood glucose < 6 mmol/L were recruited into the study. Health status was confirmed by the subject’s General Practitioner and medical exclusion criteria were: Diabetes, Kidney disease, Hepatic disease, Gout, Food allergy, Psychiatric disorders, Severe gastrointestinal disorders, Thromboembolic or Coagulation disease, Alcohol or Substance abuse, Eating disorders and Unregulated thyroid disease. The average baseline characteristics of all 18 subjects were: age 49 ± 12 years (range 21–70), BMI 36.6 ± 5.8 (range 26.5–51.7) and basic metabolic rate (BMR) 9.0 ± 1.6 MJ/day (range 6.7–12.7). BMR was measured in each fasted volunteer and used to calculate the individual energy requirements. The study is focussed on men to minimize variability as the number of participants was restricted by the complexity of the dietary design. Further details on the study volunteers and study design can be found in [18]. Medication exclusion criteria were: Orlistat, Rimonabant, Sibutramine, Oral antidiabetics including insulin, Digoxin, Anti-arrhythmics, Antidepressants, Anticoagulants and no antibiotics or drugs known to influence the faecal microbiota were taken immediately prior to or during the course of the study. The drugs reported by at least one subject on the study include: Amlodipine, Aspirin, Atenolol, Becotide (inhalers), Bendroflumethiazide, Diclofenac, Diclofex, Enalipril, Felodipine, Ibuprofen, Liptor, Statin, Tamsulosin hydrochloride, Thyroxine, Tramadol, Trazodone, Valstatin and Ventolin (inhalers). Ethical approval was granted by the North of Scotland Research Ethics Committee and all subjects provided informed signed consent. All volunteers were asked not to change their lifestyle for the duration of the study and there were no drop-outs during the study.

### Dietary intervention

There were four periods of dietary intervention, with all food supplied in cooked (breakfast) or ready-to-eat form (lunch and dinner). All food was prepared in-house and weighed by the kitchen research staff at the Human Nutrition Unit (HNU). Any leftover food that was not consumed by study subjects was collected and weighed to the nearest 0.1 g. All subjects (*n* = 18) first received a body weight maintenance diet (M, fed to 1.5× Basic Metabolic Rate, BMR) for 1 week before being randomised for consumption of three weight loss diets for 10 days each in a cross-over design. The weight loss diets were: a normal protein weight loss diet (NPWL, 15% of energy from protein, 55% from carbohydrate), a normal protein weight loss diet enriched with free amino acids and moderate amounts of carbohydrate (NPAAWL, 15% of energy from protein, 15% from free AA, 40% from carbohydrate) or a high protein weight loss diet containing moderate amounts of carbohydrate (HPWL, 30% of energy from protein, 40% from carbohydrate). The free amino acid profile of the NPAA diet matched that of beef and chicken [18]. All weight loss diets were fed at 1× BMR as three meals per day and prepared on a 5 days rotation menu. All subjects consumed all three weight loss diets in a random order for 10 days. No washout period was observed between the different diets as the faecal microbiome and metabolome have been shown to adapt to dietary changes within 1–2 days [19].

### Analysis of dietary intakes

Using the HNU kitchen record and the subjects’ food diaries, daily nutrient intakes for each subject were determined by trained staff using the WinDiets Nutritional Analysis Software Suite version 1.0 (The Robert Gordon University, Aberdeen, UK), a computerised version of McCance and Widdowson’s the composition of foods [20]. These individual daily intakes of macronutrients (carbohydrate, fat and protein), dietary fibre, non-starch polysaccharides, sugar, starch and total energy were then collated for analysis of statistical comparisons between the various diets (summarised in Table 1). For more detailed analysis of nutrient intake, exact food and nutrient intakes 4 days prior to each faecal sample collection were analysed and averages of 4 days were then correlated with faecal metabolites. Specific intakes of individual food items (e.g. meats) were extracted from the recipe sheets and the protein content of meat products was estimated as 25% of meat weight, based on a weighted average of the ingredients from the McCance and Widdowson’s food composition tables for cooked products [20]. The food group of red meat contained fresh and processed products of beef (steak, mince, topside, rump steak, burgers, sausages) and pork (roast, fillet, loin chops, sausages, ham, bacon). White meat and fish included chicken (breast, nuggets, sausages), turkey, tuna (canned in brine), prawns and haddock (in batter). The other main sources of protein were dairy products and eggs. Dietary fibre (expressed as non-starch polysaccharides) derived mainly from wholegrain bread and pasta, legumes and vegetables.

**Table 1:** Average nutrient and food intakes of 18 volunteers on each study diet

	*M*	NPWL	NPAAWL	HPWL
Energy^1^				
MJ/day	13.06 ± 1.44	9.02 ± 1.13	8.97 ± 1.07	8.82 ± 1.12
Kcal/day	3118.65 ± 343.13	2154.27 ± 270.14	2143.47 ± 256.57	2105.96 ± 268.26
Fat^1^				
g/day	106.07 ± 11.58	73.23 ± 9.27	72.92 ± 8.90	71.62 ± 8.90
Total carbohydrates^1^				
g/day	448.43 ± 49.77	308.61 ± 24.52	218.74 ± 25.76	219.37 ± 30.87
Starch^1^				
g/day	233.35 ± 26.29	167.18 ± 14.84	97.87 ± 15.84	104.60 ± 15.52
Sugar^1^				
g/day	214.70 ± 25.55	140.16 ± 10.20	119.65 ± 11.48	112.92 ± 14.57
Dietary fibre^1^				
g/day	31.83 ± 3.42	28.50 ± 3.39	19.92 ± 2.68	18.13 ± 2.39
NSP^1^				
g/day	25.49 ± 3.77	26.63 ± 3.39	18.16 ± 2.34	18.29 ± 2.26
Total protein^1^				
g/day	115.17 ± 12.58	79.54 ± 9.97	155.55 ± 18.04^2^	153.06 ± 19.71
Protein from all meat^3^				
g/day	30.35 ± 3.47	25.35 ± 3.03	33.52 ± 4.09	98.97 ± 13.72
All meat^4^				
g/day	121.40 ± 13.86	101.41 ± 12.10	134.10 ± 16.34	395.86 ± 54.88
Red meat^4^				
g/day	53.10 ± 8.52	50.32 ± 5.58	66.88 ± 12.81	158.38 ± 28.06
White meat and fish^4^				
g/day	68.30 ± 11.78	51.09 ± 6.98	67.21 ± 8.31	237.48 ± 32.03
Processed meat^4^				
g/day	27.29 ± 5.45	45.64 ± 7.27	61.44 ± 16.12	147.86 ± 27.99

Dietary components are given as mean intake over 4 days prior to faecal collection and average of all volunteers (*n* = 18) on each diet ± SD

^1^All nutrient intakes were calculated individually using WinDiets software

^2^Total protein includes addition of free amino acids (60 g/day) for the normal protein + amino acid weight loss diet (NPAAWL) to match total protein content of high protein weight loss diet (HPWL)

^3^The protein content of meat products was estimated as 25% of meat weight

^4^Specific intakes of individual foods (all meat, red meat, white meat and fish, processed meat) were extracted from the recipe sheets

### Faecal sample preparation

Freshly voided faecal samples provided by all volunteers were maintained at 4 °C for no longer than 5 h prior to processing. Each sample was mixed in a stomacher (Seward; Bury St Edmunds, UK), centrifuged (50,000*g*; 12 °C; 2 h) and supernatant was collected as faecal water and stored at − 80 °C until analyses. All faecal water samples were defrosted on ice prior to chemical analysis.

### Determination of short chain fatty acids and ammonia in faecal waters

Short chain fatty acids were determined using capillary gas chromatography [21]. The lower limit of reliable detection of each product was 0.2 mmol/L. Ammonia concentrations were analysed in 30-fold diluted faecal waters following reaction with sodium phenate and sodium hypochlorite by measuring absorbance of the indophenol product at 625 nm [22].

### Analysis of apparent total *N*-nitroso compounds (ATNC) in faecal waters

Faecal water samples were diluted 10-fold with distilled water and the content of *N*-nitroso compounds analysed by measuring the chemical release of nitric oxide detected on a thermal energy analyser. Concentrations were calculated by comparing the thermal response of a faecal water sample to the response of an *N*-nitrosodipropylamine standard (16.6 µg/mL) and values were expressed as apparent total *N*-nitroso compounds (ATNC) ng/mL faecal water sample.

### Analysis of metabolites in faecal waters

Faecal water samples were thawed to 4 °C and 80 µl transferred to an Eppendorf tube. Internal standard 1 (^13^C-labelled benzoic acid; 80 µL), internal standard 2 (4,7,8-tri-MelQx; 80 µL) and methanol (160 µL) were added. The samples were mixed, centrifuged (12,500×*g*; 5 min; 4 °C) and the supernatant analysed by LC–MS. Liquid chromatography separation of the metabolites produced was performed on an Agilent 1100 HPLC system (Agilent Technologies, Wokingham, UK) using a Zorbax Eclipse 5 µm, 150 mm × 4 mm column (Agilent Technologies). Three different gradients were used to separate the different categories of metabolites and the mobile phase solvents in each case were water containing 0.1% acetic acid and acetonitrile containing 0.1% acetic acid. In all cases the flow rate was 300 µL/min with an injection volume of 5 µL. The LC eluent was directed into, without splitting, an ABI 3200 triple quadrupole mass spectrometer (Applied Biosystems, Warrington, UK) fitted with a Turbo Ion Spray™ (TIS) source. For LC methods 1 and 2, the mass spectrometer was run in negative ion mode with the following source settings: ion spray voltage − 4500 V, source temperature 400 °C, Gases 1 and 2 set at 15 and 40, respectively, and the Curtain Gas set to 10. For LC method 3, the mass spectrometer was run in positive ion mode with the following source settings; ion spray voltage 5500, source temperature 400 °C, Gases 1 and 2 set at 14 and 40, respectively, and the Curtain Gas set at 10. All the metabolites were quantified using multiple reaction monitoring. Standard solutions (10 ng/µL) for all analytes were prepared and pumped directly via a syringe pump. The ion transitions for each of the analytes were determined based upon their molecular ion and a strong fragment ion. For several categories of compounds, it was inevitable that their molecular ion and fragment ion would be the same, but this was overcome by their differing elution times. Their voltage parameters, declustering potential, collision energy and cell entrance/exit potentials were optimised individually for each analyte.

### Statistical analysis

Power calculations indicated that with 18 volunteers a treatment effect of at least 20% could be detected with a power of 90% at the 5% significance level, assuming a within-volunteer spread of 25%.

Data were analysed by analysis of variance with volunteer as a random effect and diet as a fixed effect. Initially, the effect of order and its interaction with diet were included in the analyses but these were found to be non-significant and were therefore omitted from subsequent analyses. When the effect of diet was significant (*P* < 0.05), means were compared with post hoc *t* test. When the assumption of constant variance (and normality) was not met, data were log-transformed before analysis. This was the case for the phytochemicals, lactate, lactate%, ATNC and other protein metabolites. Zeros were replaced 0.5× the lowest non-zero value. For log-transformed data, the backtransformed (geometric) means and their corresponding 95% confidence intervals are presented. When more than 15% (more than ten samples out of 72 samples) of the samples were zero, non-parametric Friedman test was conducted instead. When the effect of diet was significant (*P* < 0.05), means were compared with Wilcoxon signed rank test. These data are presented as their mean and the number of zero samples out of the total samples tested. Linear relationships between variables were investigated with random effects linear regression, where volunteer was regarded as random. Log-transformation was applied to the same set as variables as mentioned above and only metabolites for which less than 15% of the samples were zero were included. All data were analysed with Genstat 17th Edn Release 17.1 (VSN International Ltd, Hemel Hempstead, Herts, UK). A *P* value < 0.05 was regarded as statistically significant.

## Results

### Nutrient and food intakes and weight loss on each study diet

**Fig. 1:**
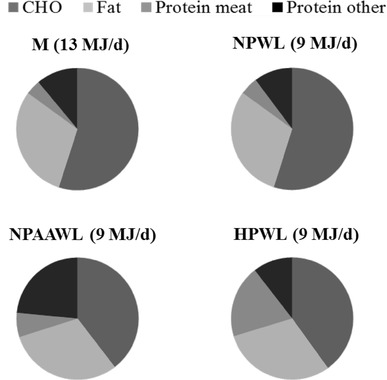
Diet composition summarised for the four study diets. Dietary components are given as percentage of total energy intake over 4 days prior to faecal collection and average of all 18 volunteers on each diet. *CHO* carbohydrate

The average scaled M diet for the volunteers was 13 MJ energy intake and contained normal proportions of macronutrients (Fig. 1). All weight loss diets were based on a reduced average energy intake (average 9 MJ/day). The NPWL diet comprised macronutrients in similar proportion to the M diet, but at decreased daily amounts. The NPAAWL diet was calculated to provide an increased protein intake in form of free amino acids which are expected to be rapidly absorbed in the small intestine. This increase was balanced by decreased carbohydrate intake from 448 to 309 g/day to make the weight loss diets iso-energetic (Table 1). The HPWL diet had a similarly decreased carbohydrate content compared to NPAAWL, but with all protein derived from normal food sources (meat, dairy and plant protein). Mean dietary fibre intake was 32 g/day (M), 29 g/day (NPWL), 20 g/day (NPAAWL) and 18 g/day (HPWL). All subjects maintained their average baseline body weight (117.2 kg) on the M diet (116.4 kg) but significantly (*P* < 0.05) decreased the body weight on all weight loss diets (112.5 kg on NPWL, 112.15 kg on NPAAWL and 112.36 kg on HPWL diets) [18]. Effect of diet on faecal SCFA.

The faecal short chain fatty acid butyrate was decreased (*P* < 0.028) in faeces of individuals on HPWL diet compared to M (19.16 versus 28.55 mM or 15.89 versus 19.43% of total SCFA, respectively; Table 2). Faecal propionate, iso-butyrate, iso-valerate and valerate (as % of total SCFA) were increased (*P* < 0.043) in faeces of individuals consuming NPAAWL or HPWL diets when compared to M diet, but no such increase was observed when volunteers consumed the NPWL diet.

**Table 2:** Faecal pH and short chain fatty acids in faecal waters

mmol/L	MTD	NPWL	NPAAWL	HPWL	SED^1^	*P* diet^1^
pH	6.69^a^	6.54^a,b^	6.43^b^	6.75^a^	0.12	0.043
Total SCFA	140.44	125.87	120.47	112.53	12.29	0.15
Acetate	77.03	68.09	64.88	61.11	6.77	0.12
Propionate	24.58	22.57	23.00	21.80	2.25	0.66
Butyrate	28.55^a^	25.72^a,b^	22.06^a,b^	19.16^b^	3.29	0.034
Isobutyrate	2.60	2.57	2.72	2.90	0.32	0.71
Isovalerate	2.20	2.26	2.49	2.69	0.31	0.39
Total BCFA	4.80	4.83	5.21	5.59	0.63	0.55
Valerate	3.46	3.11	3.45	3.41	0.37	0.74
Lactate	1.76 (1.36; 2.28)	1.49 (1.15;1.92)	1.61 (1.24;2.08)	1.43 (1.11; 1.85)	na	0.39^2^
**% of total SCFA**						
Acetate	55.75	55.23	53.63	54.90	1.30	0.420
Propionate	17.21^a^	17.89^a,b^	19.17^b,c^	19.72^c^	0.72	0.004
Butyrate	19.43^a^	19.06^a^	18.19^a^	15.89^b^	1.01	0.005
Isobutyrate	1.89^a^	2.12^a,b^	2.35^b,c^	2.59^c^	0.21	0.015
Isovalerate	1.62^a^	1.90^a,b^	2.17^b,c^	2.39^c^	0.23	0.009
Total BCFA	3.51^a^	4.02^a,b^	4.52^b,c^	4.98^c^	0.44	0.011
Valerate	2.50^a^	2.49^a^	2.89^b^	2.94^b^	0.19	0.028
Lactate	1.31 (0.95; 1.82)	1.24 (0.89;1.72)	1.38 (1.00;1.92)	1.39 (1.01; 1.93)	na	0.87^2^

Values are presented as means of 18 volunteers

^1^Analysed by ANOVA with volunteer as random effect and diet as fixed effect. SED is standard error of the difference for comparing diet means. When the effect of diet was significant (*P* < 0.05), means were compared with post hoc *t* test. Means not sharing a superscript (a,b,c)  are significantly (*P* < 0.05) different

^2^Data were log-transformed before analysis. Presented are backtransformed means and corresponding 95% confidence intervals

### Effect of diet on phenolic compounds in faecal waters

Among the benzoic acid derivatives, several compounds were decreased in faeces of subjects consuming HPWL or NPAAWL diets compared to M (salicylic acid, gentisic acid, 2,6-dihydroxybenzoic acid, 3,5-dihydroxybenzoic acid, all *P* < 0.043), whereas others increased on the WL diets compared to M (protocatechuic acid, m-anisic acid, gallic acid, vanillic acid, syringic acid, all *P* < 0.027). Several benzaldehydes also increased on WL diets (p-hydroxybenzaldehyde, protocatachaldehyde, 3,4,5-trihydroxybenzaldehyde, syringin, all *P* < 0.034), whereas most acetophenones decreased on the HPWL diet (all *P* < 0.036, Table 3). Cinnamic acid and two cinnamic acid derivatives (ferulic and sinapic acid) as well as 4-hydroxy-3-methoxyphenylpropionic acid were lower in faecal waters from subjects consuming WL diets compared with M (all *P* < 0.021, Table 4). On the other hand 4-hydroxymandelic acid, 3,4-dihydroxymandelic acid and 4-hydroxyphenylpyruvic acid were increased on one or more of the WL diets compared to M (all *P* < 0.047). Faecal phenylacetic acids and most other metabolites were not significantly altered as a result of diet (Online Resource 1 and 2). Phenol was below the limit of detection in 69 of 72 faecal water samples tested. Several faecal metabolites were detected at very low concentrations and were absent in faecal samples of some volunteers, indicating inter-individual variation in colonic substrate metabolism [23].

**Table 3:** Concentrations of benzoic acids, benzaldehydes, benzenes and acetophenones in faecal waters

Name	*M*	NPWL	NPAAWL	HPWL	*P* diet^1^
**Benzoic acids (ng/mL)**					
Salicylic acid	20.94^a^ (15.17; 28.91)	13.79^b^ (9.99; 19.03)	13.63^b^ (9.87; 18.81)	11.00^b^ (7.97; 15.19)	0.002
Gentisic acid	183.11^a^ (108.80; 308.18)	148.01^a,b^ (97.94; 249.11)	92.69^b^ (55.08; 156.01)	90.16^b^ (53.57; 151.74)	0.018
2,6-Dihydroxybenzoic acid	6.89^a^ (4.33; 10.94)	3.40^b^ (2.14; 5.40)	2.88^b,c^ (1.81; 4.57)	2.11^c^ (1.33; 3.35)	0.000
Protocatechuic acid	42.74^a^ (30.75; 59.40)	122.65^b^ (88.24; 170.48)	146.82^b^ (105.63; 204.07)	106.08^b^ (76.32; 147.45)	0.000
3,5-Dihydroxybenzoic acid	221.51^a^ (174.73; 280.83)	220.42^a^ (173.86; 279.44)	135.57^b^ (106.94; 171.88)	148.02^b^ (116.76; 187.65)	0.000
o-Anisic acid	11.30 (8/18)	30.78 (5/18)	33.22 (6/18)	28.51 (7/18)	0.084^2^
m-Anisic acid	0.96^a^ (13/18)	3.47^b^ (7/18)	3.89^b^ (5/18)	3.14^b^ (6/18)	0.007^2^
Gallic acid	12.05^a^ (7.89; 18.41)	36.34^b^ (23.79; 55.52)	36.18^b^ (23.68; 55.27)	31.81^b^ (20.82; 48.60)	0.000
Vanillic acid	45.29^a^ (31.39; 65.35)	100.73^b^ (69.81; 145.34)	118.93^b^ (82.42; 171.60)	91.24^b^ (63.24; 131.65)	0.000
Syringic acid	22.96^a^ (14.11; 37.36)	59.81^b^ (36.76; 97.31)	42.50^b,c^ (26.12; 69.15)	34.43^a,c^ (21.16; 56.02)	0.002
3,4-Dimethoxybenzoic acid	32.29 (13/18)	97.97 (12/18)	99.18 (10/18)	88.26 (9/18)	0.099^2^
**Benzaldehydes (ng/mL)**					
p-Hydroxybenzaldehyde	71.38 ^a^(50.35; 101.20)	86.96^a,b^ (61.34; 123.29)	107.75 ^b^ (76.00; 152.77)	122.22^b^ (86.21; 173.28)	0.017
Protocatachaldehyde	8.30^a^ (6.44; 10.70)	12.59^b^ (9.77; 16.23)	16.80^c^ (13.03; 21.65)	15.81^b,c^ (12.27; 20.38)	0.000
3,4,5-Trihydroxybenzaldehyde	2.18^a^ (12/18)	16.66^b^ (1/18)	15.29^b^ (2/18)	15.77^b^ (2/18)	0.000^2^
Syringin	4.47^a^ (3.13; 6.38)	8.89^b^ (6.23; 12.70)	6.75^b^ (4.73; 9.65)	6.58^b^ (4.60; 9.39)	0.004
**Benzenes (ng/mL)**					
Catechol	63.60^a^ (47.52; 85.12)	94.84^b^ (70.68; 126.93)	86.01^b,c^ (64.23; 115.12)	68.12^a,c^ (50.90; 91.17)	0.025
Resorcinol	10.66 (9/18)	10.64 (13/18)	4.60 (14/18)	4.58 (13/18)	0.077^2^
**Acetophenones (ng/mL)**					
4-Hydroxyacetophenone	1.68^a^ (6/18)	0.64^b^ (12/18)	1.26^a,b^ (7/18)	0.80^b^ (10/18)	0.034^2^
4-Hydroxy-3-methoxyacetophenone	2.42^a^ (8/18)	0.95^a,b^ (12/18)	0.62^b^ (13/18)	0.40^b^ (15/18)	0.024^2^
4-Hydroxy-3,5-dimethoxyacetophenone	10.44^a^ (4/18)	7.37^a^ (6/18)	5.78^a,b^ (8/18)	3.00^b^ (12/18)	0.014^2^
3,4,5-Trimethoxyacetophenone	348.77 (237.46; 512.27)	546.74 (372.24; 803.03)	353.65 (240.78; 519.43)	466.94 (317.91; 685.83)	0.060

^1^Analysed by ANOVA with volunteer as random effect and diet as fixed effect. When the effect of diet was significant (*P* < 0.05), means were compared with post hoc *t* test. Means not sharing a superscript (a,b,c)  are significantly (*P* < 0.05) different. Data were log-transformed before analysis. Presented are backtransformed means (based on 18 volunteers) and corresponding 95% confidence intervals

^2^Analysed by Friedman non-parametric test. When the effect of diet was significant (*P* < 0.05), means were compared with Wilcoxon signed rank test. Means not sharing a superscript (a, b, c) are significantly (*P* < 0.05) different. Presented are the means and the number of samples that were zero, out of 18 samples per diet

**Table 4:** Concentrations of cinnamic, phenylpropionic and phenylacetic acids, indoles, heterocyclic amines, apparent *N*-nitroso compounds (ATNC) and nitrite in faecal waters (ng/mL) presented as geometric means

Name	MTD	NPWL	NPAAWL	HPWL	*P* diet^1^
Cinnamic acid (ng/mL)	21.39^a^ (14.16; 32.30)	14.75^a,b^ (9.77; 22.28)	11.91^b^ (7.89; 18.00)	16.37^a,b^ (10.84; 24.72)	0.049
Ferulic acid (ng/mL)	566.70^a^ (299.40; 1072.65)	402.14^a,b^ (212.46; 761.17)	231.61^b,c^ (122.36; 438.38)	133.55^c^ (70.56; 252.78)	0.000
Sinapic acid (ng/mL)	54.77^a^ (30.59; 98.07)	27.42^b^ (15.31; 49.09)	24.20^b^ (13.51; 43.33)	16.82^b^ (9.39; 30.12)	0.002
4-Hydroxy-3-methoxyphenylpropionic acid (ng/mL)	1193.94^a^ (824.15; 1729.65)	867.63^a^ (598.91; 1256.94)	465.20^b^ (321.11; 673.93)	411.83^b^ (284.27;596.61)	0.000
**Others (ng/mL)**					
4-Hydroxymandelic acid	26.61^a^ (16/18)	122.97^b^ (12/18)	97.94^a,b^ (13/18)	89.36^a,b^ (13/18)	0.038^2^
3,4-Dihydroxymandelic acid	134.25^a^ (110.68; 162.83)	180.33^b^ (148.67; 218.72)	162.82^a,b^ (134.24; 197.48)	163.20^b^ (134.55;197.95)	0.028
4-Hydroxyphenylpyruvic acid	1010.28^a^ (11/18)	1639.17^a^ (8/18)	1793.33^a^ (7/18)	2421.67^b^ (5/18)	0.031^2^
4-Hydroxyphenyllactic acid	974.71^a^ (674.58; 1408.39)	775.88^a,b,c^ (536.97; 1121.09)	578.90^c^ (400.65; 836.47)	855.45^a,b^ (592.04; 1236.06)	0.043
P-cresol (ng/ul)	10.83 (7.56; 15.52)	12.09 (8.44; 17.32)	16.06 (11.21; 23.00)	16.34 (11.41; 23.40)	0.059
**Indoles (ng/mL)**					
Indole-3-propionic acid	385.21 (268.63; 552.37)	390.74 (272.49; 560.30)	271.22 (189.14; 388.91)	268.55 (187.27; 385.09)	0.054
Indole-3-carboxylic acid	20.18^a^ (15.67; 25.98)	14.30^b^ (11.11; 18.41)	14.16^b^ (11.00; 18.23)	16.98^a,b^ (13.19; 21.85)	0.021
Indole-3-methyl	28.83 (10/18)	20.32 (11/18)	24.64 (10/18)	29.93 (10/18)	0.093^2^
Indole-3-lactic acid	18.79^a^ (13.50; 26.15)	14.85^a,b^ (10.67; 20.67)	11.04^b^ (7.93; 15.37)	15.73^a^ (11.30; 21.89)	0.020
**Heterocyclic amines and ATNC (ng/mL)**					
2-Amino-3methylimidazo[4,5-f]quinoline	6.85^a^ (5.35; 8.76)	9.20^b^ (7.19; 11.77)	10.10^b^ (7.90; 12.92)	10.23^b^ (8.00; 13.08)	0.006
2-Amino-3,4-dimethylimidazo[4,5-f]quinoline	3.08^a^ (2.32; 4.08)	4.11^b^ (3.10; 5.45)	4.33^b^ (3.27; 5.75)	4.71^b^ (3.55; 6.24)	0.024
2-Amino-3,8-dimethylimidazo[4,5-f]quinoxaline	5.30^a^ (3.80; 7.39)	7.28^a,b^ (5.22; 10.15)	7.89^b^ (5.66; 11.00)	8.52^b^ (6.11; 11.88)	0.033
2-Amino-3,7,8-timethylimidazo[4,5-f]quinoxaline	11.92^a^ (9.97; 14.26)	13.61^a,b^ (11.38; 16.28)	14.13^a,b^ (11.82; 16.91)	15.76^b^ (13.17; 18.85)	0.024
2-Amino-1-methyl-6-phenylimiazo[4,5-b]pyridine	5.84^a^ (5.13; 6.64)	7.19^b^ (6.32; 8.18)	8.06^b,c^ (7.09; 9.17)	8.59^c^ (7.55; 9.77)	0.000
2-Amino-1,6-dimethylimiazo[4,5-b]pyridine	4.64^a^ (3.74; 5.77)	6.25^b^ (5.03; 7.76)	6.53^b^ (5.26; 8.11)	6.66^b^ (5.36; 8.28)	0.006
Apparent total *N*-nitroso compounds	302.76^a^ (227.18; 403.49)	415.56^b^ (311.82; 553.81)	423.89^b,c^ (318.07; 564.92)	563.21^c^ (422.61; 750.59)	0.001

^1^Analysed by ANOVA with volunteer as random effect and diet as fixed effect. When the effect of diet was significant (*P* < 0.05), means were compared with post hoc *t* test. Means not sharing a superscript (a,b,c)  are significantly (*P* < 0.05) different. Data were log-transformed before analysis. Presented are backtransformed means (based on 18 volunteers) and corresponding 95% confidence intervals

^2^Analysed by Friedman non-parametric test. When the effect of diet was significant (*P* < 0.05), means were compared with Wilcoxon signed rank test. Means not sharing a superscript (a, b, c) are significantly (*P* < 0.05) different. Presented are the means and the number of samples that were zero, out of 18 samples per diet

### Effect of diet on indoles, heterocyclic amines and apparent *N*-nitroso compounds (ATNC)

Only minor changes were observed in indoles in faecal waters (Table 4), whereas heterocyclic amines were increased on the HPWL diet compared to M (all *P* < 0.006), and several of these were also increased on either the NPWL or the NPAAWL diet (*P* < 0.045). Faecal ATNC were also affected by diet (*P* = 0.001), with concentrations in faeces increased by all three weight loss diets compared to M (*P* < 0.031). ATNC in faeces of individuals consuming NPWL diet were lower compared to HPWL diets (*P* = 0.038).

### Direct interactions between dietary components and faecal metabolites

As expected from the experimental design, dietary intakes of carbohydrates (total carbohydrate, starch, sugar, dietary fibre, non-starch polysaccharides or NSP) were strongly interrelated, as were dietary protein intakes (total protein, protein from red meat, protein from white meat, protein from all meat). Furthermore, faecal SCFA were positively associated with ferulic acid (*r* = 0.39, *P* < 0.001 where *r* is simple correlation coefficient and the *P* value is from random effects regression) and negatively associated with faecal pH (*r* = − 0.56, *P* < 0.001), whereas faecal branched chain fatty acids (BCFA) were associated with phenylacetic acid (*r* = 0.86, *P* < 0.001), p-cresol (*r* = 0.83, *P* < 0.001), ammonia (*r* = 0.52, *P* < 0.001) and ATNC (*r* = 0.42, *P* < 0.001).

**Fig. 2:**
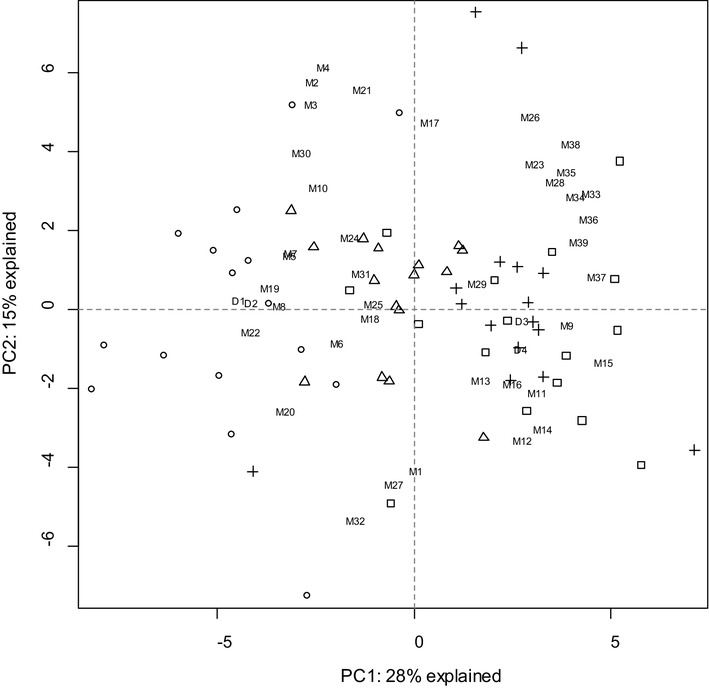
PCA plot for all metabolites for which ANOVA showed a significant effect of diet, or for which the random effects regression showed a significant relationship between dietary components and metabolic products. Also included are four representative dietary intakes. All data were log-transformed except for dietary intakes, SCFA concentrations and pH and were centred within volunteer. Results are presented for samples from volunteers on M diet (circle); NPWL diet (triangle); NPAAWL diet (plus) and HPWL diet (square). Major dietary intakes are depicted as D1 (carbohydrate), D2 (dietary fibre), D3 (protein) and D4 (meat). Faecal metabolites are depicted as M1–M39, full chemical names are displayed in Fig. 3

Principal component analysis (PCA) was used to further explore the diet-associated pattern of the faecal metabolome in volunteer samples (Fig. 2). Samples from volunteers on M diet were separated from those on HPWL diet, whereas samples from intermediate diets (NPWL, NPAAWL) were more dispersed. Samples from volunteers on M diet clustered around dietary carbohydrate intake and carbohydrate-related fermentation products (ferulic acid as well as butyrate, acetate and total SCFA). Samples from volunteers consuming HPWL diet clustered around dietary protein and meat intake as well as some products of amino acid fermentation (phenylacetic acid, indole-3-acetic acid, p-cresol) and meat-derived carcinogens (heterocyclic amines and ATNC).

**Fig. 3:**
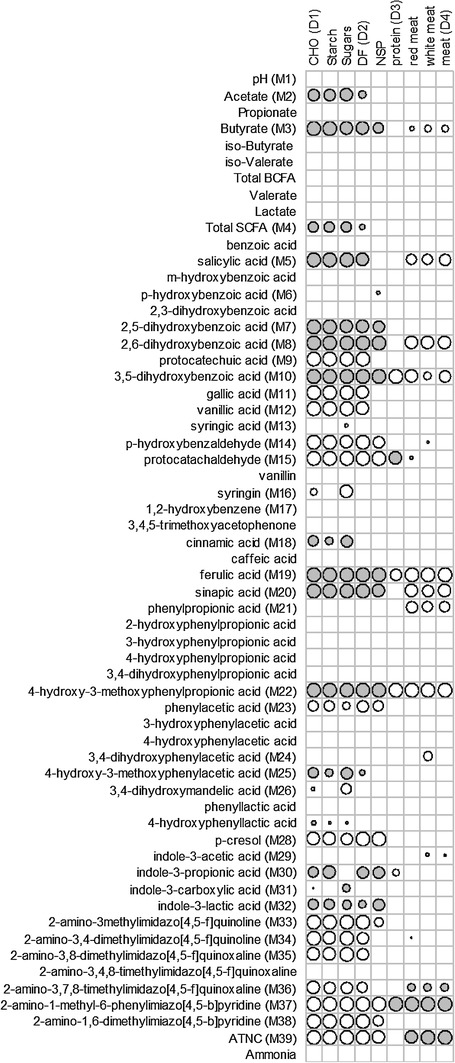
Associations between dietary intakes and faecal metabolites. Grey circle significant (*P* < 0.05) positive relationship, white circle significant (*P* < 0.05) negative relationship, bigger circle means more significant. Blank entries indicate non-significant association. Based on random effects regression of metabolite (dependent variable) on dietary component (explanatory variable), with volunteer as random effect. All metabolites were log-transformed before analysis with the exception of pH, ammonia and the SCFA concentrations (except for lactate, which was log-transformed). Only metabolites for which no more than ten samples (out of a total of 72) were zero or at the limit of detection were analysed

Interactions between dietary components and faecal metabolites were also explored by correlation analysis (Fig. 3). Dietary carbohydrate appeared the main driver of colonic fermentation as most faecal metabolites were significantly associated with carbohydrate intakes including total carbohydrate, starch, sugar, dietary fibre and NSP (28/60), some were associated with both carbohydrate and protein intakes (10/60) and only one metabolite (phenylpropionic acid) was solely associated with meat protein intake, but not with total protein intake. All carbohydrate components were positively associated with many of the carbohydrate-related faecal metabolites including acetate, butyrate, total SCFA, salicylic acid, gentisic acid, 2,6-dihydroxybenzoic acid, 3,5-dihydroxybenzoic acid, ferulic acid, sinapic acid and 4-hydroxy-3-methoxyphenylproprionic acid. Carbohydrate intakes were also negatively associated with some benzoic acids (protocatechuic acid, gallic acid, vanillic acid) as well as some metabolites related to colonic amino acid fermentation (phenylacetic acid, p-cresol) and meat intake (heterocyclic amines, ATNC).

Dietary total protein and meat protein were negatively associated with faecal concentrations of 3,5-dihydroxybenzoic acid, ferulic acid and 4-hydroxy-3-methoxyphenylproprionic acid. Furthermore, dietary meat protein was negatively associated with butyrate, salicylic acid, 2,6-dihdroxybenzoic acid, sinapic acid and phenylpropionic acid. None of the protein intakes was associated with any amino acid-derived fermentation products (BCFA, phenylacetic acid, 4-hydroxyphenylacetic acid, indole-3-acetic acid). Strong positive correlations were found between 2-amino-1-methyl-6-phenylimiazo[4,5-b]pyridine and both dietary total protein and meat protein and between ATNC and dietary meat protein.

## Discussion

Diet is widely recognised as main driver of colonic fermentation (microbial metabolome), mainly through providing fermentable substrate rather than altering bacterial community structure [24]. The current study aims to identify the most important dietary contributors to colonic fermentation in subjects consuming controlled weight loss diets that differ in carbohydrate, fibre and protein content. The study is limited to a small number (*n* = 18) of obese or overweight male subjects, but at the same time this design facilitates very rigorous control of dietary intakes over an extended period (37 days in total). Diets were designed to contain dietary fibre at either the current UK dietary recommendations of 30 g/day (for M and NPWL diets) or the UK average intake of 18 g/day (for NPAAWL and HPWL diets) [1]. At the same time, dietary total protein varied substantially (115, 79, 156 and 153 g/day for M, NPWL, NPAAWL and HPWL diets, respectively). All three weight loss diets resulted in similar weight loss.

The most pronounced dietary change occurred between the balanced M diet and the HPWL diet and this change induced a substantial shift in faecal metabolite profiles. Most importantly, the main fibre-derived beneficial metabolites were decreased in HPWL diets including butyrate, ferulic acid, sinapic acid and 4-hydroxy–3-methoxyphenylpropionic acid as well as salicylic and gentisic acid. These changes are more pronounced than reported in a similar study comparing M and HPWL diets (only butyrate was decreased when NSP fibre intake was decreased from 22 to 13 g/day, [17]). These compounds are believed to exert potent antioxidant effects and other beneficial health effects, and their increased faecal concentrations might reflect intake of fruit and vegetables [25]. At the same time, meat-related potential carcinogens including several HCA and ATNC were increased in faeces of subjects consuming HPWL diets. However, this increase was less pronounced than reported previously, possibly due to the relatively high amount of fibre present in HPWL diet compared to the published study [17]. Similarly, branched chain fatty acids, p-cresol and 4-hydroxyphenylpyruvic acid increased in faeces of subjects on HPWL diets as did potential amino-acid metabolites p-hydroxybenzaldehyde and protocatachaldehyde. Other metabolites linked to the fermentation of aromatic amino acids [9] such as indoles, phenylacetic acid and 4-hydroxyphenylacetic acid as well as ammonia remained unchanged by diet. Similarly, Russell et al. [17] found BCFA and phenylacetic acid increasing, whereas other metabolites were unchanged. These findings point towards potentially increased colonic amino acid fermentation during HPWL diet period, although not all relevant metabolites respond.

In comparison, the shift from M to NPAAWL diet also induced significant changes in the faecal metabolome. With the exception of butyrate, all other changes were comparable to the ones induced by HPWL diet. This finding is important, especially when meat-derived carcinogenic compounds are concerned. Despite a 2.9-fold lower meat intake on the NPAAWL diet compared to HPWL, the faecal concentrations of heterocyclic amines and ATNC were not significantly different. We found that consuming a HPWL diet resulted in a 1.9-fold increase in ATNC compared to M diet, while consuming NPAAWL led to a 1.4-fold increase. Possibly, the amount of dietary fibre in both HPWL and NPAAWL diets (18 g/day) was sufficient to counteract an increase in colonic ATNC production observed previously (3.6-fold increase, [17]). Other factors such as cooking and preservation method as well as Vitamin C intake might have also influenced the faecal levels of meat-related carcinogens in the NPAAWL diet [12].

When subjects consumed a balanced NPWL diet, several differences in faecal metabolome were observed compared to M. Several phytochemical-derived metabolites (gallic acid, vanillic acid, syringic acid, syringin) were increased in NPWL diets. At the same time some meat-related carcinogens also increased which probably reflects the increase in processed meat on NPWL diets compared to M. However, many of these changes were less pronounced then in HPWL diet. Taken together, NPWL diet appears preferable among the three weight loss diets in terms of faecal metabolite profiles as several beneficial fermentation products (butyrate, ferulic acid, its metabolites 4-hydroxy-3-methoxyphenylpropionic acid as well as syringic acid) are higher compared with the HPWL diet, while some meat-derived carcinogens (2-amino-1-methyl-6-phenylimiazo[4,5-b]pyridine, ATNC) are lower. At the same time, the extent of weight loss achieved by all three diet regimes was similar (average 4.1 kg), although hunger scores were higher for subjects on NPWL diet [18].

Correlation analysis between dietary intakes and faecal metabolites clearly indicates that dietary carbohydrates and fibre drive the most significant changes in faecal metabolite profiles. The interactions with dietary protein appear secondary as most faecal metabolites which were associated with protein are also inversely correlated with carbohydrate intakes. This is in line with the notion that most fermentable protein in the colon derives from endogenous sources rather than dietary intake [8]. Dietary non-digestible carbohydrates are the primary substrate for microbial fermentation in the human colon and only once the carbohydrate sources are depleted will protein breakdown and amino acid fermentation increase for certain microbial species. Consuming wheat-bran supplements as part of a normal diet not only increases the faecal and systemic level of ferulic acids and other beneficial carbohydrate related metabolites, but at the same time decreases amino acid fermentation products, although non-significantly [6]. Supplementing high-protein diets (meat, dairy or vegetable protein) with dietary fibre (type IV resistant starch or pectin) significantly shifts faecal fermentation towards increased SCFA and decreases amino acid fermentation [26, 27] and prevents red meat-induced pro-mutagenic lesions in rectal mucosa in humans [26]. It is therefore highly likely that delivery of larger amounts of dietary fibre in the diet could be used as a tool to prevent and offset the negative effect of increased protein consumption in terms of protein fermentation products in the colonic environment. Future studies will corroborate these findings in wider populations and specific interventions will further affirm the causality of faecal metabolomics changes.

## Conclusion

In the current study consumption of a balanced normal protein weight loss diet achieved weight loss without significantly altering the faecal metabolite profile. Consumption of a high protein diet on the other hand shifted the faecal metabolome towards a more detrimental profile. Our findings suggest that the decrease in carbohydrate and fibre content of high-protein weight loss diets contributes to these detrimental shifts and should be avoided when designing future weight loss diets.

## Electronic supplementary material

Below is the link to the electronic supplementary material.

Supplementary material 1 (DOCX 21 KB)
