# Supplementary material for: Dietary carbohydrate rather than protein intake drives colonic microbial fermentation during weight loss
Source: Eur J Nutr. 2018 Feb 20;58(3):1147–58. doi: 10.1007/s00394-018-1629-x (PMC6499751; doi:10.1007/s00394-018-1629-x)
Supplement: Supplementary file 1 — Supplementary material 1 (DOCX 21 KB) [file 394_2018_1629_MOESM1_ESM.docx]

**Table 1 Supplement**: non-significant results

| name | M | NPWL | NPAAWL | HPWL | P Diet^1^ | |
| --- | --- | --- | --- | --- | --- | --- |
| Benzoic acids |  |  |  |  |  |  |
| Benzoic acid | 1115.76 (869.55;1431.69) | 992.01 (773.10;1272.89) | 1045.51 (814.80;1341.54) | 888.56 (692.48;1140.15) | 0.32 |  |
| m-hydroxybenzoic acid | 138.25 (96.56; 197.94) | 140.23 (97.95; 200.77) | 111.95 (78.19; 160.29) | 103.02 (71.96; 147.50) | 0.23 |  |
| p-hydroxybenzoic acid | 107.99 (72.27; 161.37) | 103.89 (69.52; 155.24) | 87.95 (58.86; 131.43) | 84.47 (56.53; 126.23) | 0.54 |  |
| 2,3-dihydroxybenzoic acid | 28.18 (20.20; 39.32) | 26.73 (19.16; 37.29) | 28.04 (20.10; 39.11) | 25.05 (17.96; 34.95) | 0.88 |  |
| 2,4-dihydroxybenzoic acid | 0.37 (16/18) | 1.11 (15/18) | 0.54 (16/18) | 0.68 (15/18) | 0.80^2^ |  |
| p-anisic acid | 4.59 (11/18) | 4.09 (12/18) | 4.65 (9/18) | 2.43 (13/18) | 0.55^2^ |  |
| Benzaldehydes |  |  |  |  |  |  |
| vanillin | 7.24 (5.66; 9.26) | 8.94 (6.99; 11.44) | 8.92 (6.97; 11.41) | 8.49 (6.64; 10.86) | 0.28 |  |
| isovanillin | 0.00 | 0.00 | 0.00 | 0.00 | NA |  |
| 3-methoxybenzaldehyde | 0.00 | 0.00 | 0.00 | 0.00 | NA |  |
| 3,4-dimethoxybenzaldehyde | 2.74 (12/18) | 1.73 (12/18) | 3.65 (10/18) | 2.94 (13/18) | 0.74^2^ |  |
| 3,4,5-trimethoxybenzaldehyde | 5.29 (5/18) | 8.39 (3/18) | 6.02 (7/18) | 6.37 (4/18) | 0.62^2^ |  |
| Benzenes |  |  |  |  |  |  |
| pyrogallol | 10.50 (10/18) | 14.63 (7/18) | 8.35 (9/18) | 10.42 (11/18) | 0.50^2^ |  |
| Acetophenones |  |  |  |  |  |  |
| 3,4-dimethoxyacetophenone | 0.00 | 0.00 | 0.00 | 0.00 | NA |  |

^1^Analysed by ANOVA with volunteer as random effect and diet as fixed effect. When the effect of diet was significant (P<0.05), means were compared with post hoc t-test. Means not sharing a superscript are significantly (P<0.05) different. Data were log-transformed before analysis. Presented are backtransformed means (based on 18 volunteers) and corresponding 95% confidence intervals.

^2^Analysed by Friedman non-parametric test. No post-hoc comparisons were performed. Presented are the means and the number of samples that were zero, out of 18 samples per diet.

**Table 2 Supplement**: non-significant results

| NAME | M | NPWL | NPAAWL | HPWL | | SED | P Diet^1^ |
| --- | --- | --- | --- | --- | --- | --- | --- |
| Cinnamic acids |  |  |  |  | |  |  |
| o-coumaric acid | 0.00 (18/18) | 10.94 (16/18) | 0.00 (18/18) | 0.00 (18/18) | |  | 0.11^2^ |
| m-coumaric acid | 8.17 (17/18) | 12.84 (15/18) | 9.42 (16/18) | 30.71 (15/18) | |  | 0.42^2^ |
| p-coumaric acid | 75.69 (12/18) | 42.75 (12/18) | 40.32 (11/18) | 39.84 (12/18) | |  | 0.96^2^ |
| caffeic acid | 43.93 (26.77;72.08) | 48.55 (29.59;79.67) | 55.19 (33.63;90.56) | 43.34 (26.41;71.12) | |  | 0.75 |
| 3-methoxycinnamic acid | 0.00 (18/18) | 0.00 (18/18) | 0.00 (18/18) | 2.97 (17/18) | |  | 0.39^2^ |
| 4-methoxycinnamic acid | 0.00 | 0.00 | 0.00 | 0.00 | |  | NA |
| 3,4-dimethoxycinnamic acid | 3.95 (11/18) | 2.41 (12/18) | 2.13 (10/18) | 2.74 (11/18) | |  | 0.95^2^ |
| 3,4,5-trimethoxycinnamic acid | 0.99 (15/18) | 0.24 (16/18) | 0.63 (14/18) | 0.14 (17/18) | |  | 0.37^2^ |
| phenylpropionic acid | 17471.71 (12809.90; 23830.07) | 18218.31 (13357.29; 24848.36) | 16421.91 (12040.21; 22398.22) | 12787.11 (9375.25; 17440.63) | |  | 0.11 |
| 2-hydroxyphenylpropionic acid | 51.55 (34.25; 77.61) | 59.51 (39.53; 89.58) | 45.83 (30.45; 68.99) | 46.50 (30.89; 70.00) | |  | 0.56 |
| 3-hydroxyphenylpropionic acid | 2040.05 (1112.76; 3740.06) | 2669.84 (1456.29; 4894.68) | 2354.91 (1284.51; 4317.31) | 1434.61 (782.52; 2630.09) | |  | 0.21 |
| 4-hydroxyphenylpropionic acid | 357.21 (210.63; 605.78) | 480.78 (283.50; 815.34) | 581.59 (342.94; 986.30) | 438.40 (258.51; 743.47) | |  | 0.32 |
| 3,4-dihydroxyphenylpropionic acid | 663.77 (494.70; 890.63) | 743.58 (554.18; 997.71) | 671.50 (500.46; 900.99) | 634.52 (472.90; 851.38) | |  | 0.74 |
| 3-methoxyphenylpropionic acid | 0.00 (18/18) | 19.67 (17/18) | 2.83 (17/18) | 2.04 (17/18) | |  | 0.39^2^ |
| phenylacetic acid | 39196.36 (29658.88; 51800.82) | 43894.68 (33213.98; 58009.99) | 49556.56 (37498.18; 65492.57) | 53185.62 (40244.20; 70288.65) | |  | 0.15 |
| 3-hydroxyphenylacetic acid | 2676.93 (1536.46; 4663.95) | 3863.49 (2217.50; 6713.26) | 3062.95 (1758.02; 5336.49) | 2920.11 (1676.04; 5087.63) | |  | 0.59 |
| 4-hydroxyphenylacetic acid | 1410.00 (890.94; 2231.47) | 1634.44 (1032.75; 2586.66) | 1415.79 (894.60; 2240.64) | 1313.12 (829.72; 2078.14) | |  | 0.81 |
| 3,4-dihydroxyphenylacetic acid | 65.01 (37.23; 113.51) | 70.60 (40.43; 123.26) | 80.50 (46.11; 140.56) | 43.67 (25.01; 76.25) | |  | 0.16 |
| 4-hydroxy-3-methoxyphenylacetic acid | 470.74 (330.93; 669.61) | 365.86 (257.20; 520.42) | 331.66 (233.16; 471.77) | 310.04 (217.96; 441.02) | |  | 0.10 |
| 4-methoxyphenylacetic acid | 0.47 (14/18) | 0.34 (15/18) | 0.23 (16/18) | 0.68 (13/18) | |  | 0.21^2^ |
| Others |  |  |  |  | |  |  |
| mandelic acid | 19.42 (10/18) | 18.48 (10/18) | 23.61 (10/18) | 12.01 (13/18) | |  | 0.84^2^ |
| 3-hydroxymandelic acid | 10.96 (6/18) | 26.69 (5/18) | 29.73 (6/18) | 22.11 (5/18) | |  | 0.20^2^ |
| 4-hydroxy-3-methoxymandelic acid | 9.93 (12/18) | 9.56 (13/18) | 10.75 (15/18) | 3.97 (16/18) | |  | 0.50^2^ |
| phenylpyruvic acid | 0.00 | 0.00 | 0.00 | 0.00 | |  | NA |
| phenyllactic acid | 1066.81 (695.78; 1635.69) | 829.14 (540.77; 1271.28) | 684.83 (446.65; 1050.02) | 976.65 (636.98; 1497.46) | |  | 0.18 |
| phenol | 0.00 (18/18) | 0.76 (17/18) | 1.00 (17/18) | 2.04 (17/18) | |  | 0.39^2^ |
| indole | 4.28 (15/18) | 1.43 (17/18) | 4.85 (16/18) | 7.64 (15/18) | |  | 0.68^2^ |
| indole-3-acetic acid | 271.94 (164.48; 449.61) | 279.12 (168.82; 461.47) | 296.75 (179.48; 490.62) | 420.65 (254.43; 695.47) | |  | 0.28 |
| indole-3-acrylic acid | 0.00 | 0.00 | 0.00 | 0.00 | |  | NA |
| indole-3-carbinol | 0.00 | 0.00 | 0.00 | 0.00 | |  | NA |
| 2-amino-3,4,8-timethylimidazo [4,5-f]quinoxaline | 10.78 (9.17; 12.68) | 12.22 (10.39; 14.37) | 12.21 (10.38; 14.36) | 12.72 (10.82; 14.95) | | na | 0.22 |
| Nitrite (ng/mL) | 168.89 (6/18)^3^ | 148.61 (8/18)^3^ | 163.33 (8/18)^3^ | 187.50 (7/18)^3^ | | na | 0.52^2^ |
| Ammonia (µg/mL)^4^ | 1180.35 | 999.34 | 1182.22 | 939.14 | 167.98 | | 0.36 |

^1^Analysed by ANOVA with volunteer as random effect and diet as fixed effect. When the effect of diet was significant (P<0.05), means were compared with post hoc t-test. Means not sharing a superscript are significantly (P<0.05) different. Data were log-transformed before analysis. Presented are backtransformed means (based on 18 volunteers) and corresponding 95% confidence intervals.

^2^Analysed by Friedman non-parametric test. No post-hoc comparisons were performed. Presented are the means and the number of samples that were zero, out of 18 samples per diet.

^3^Number of samples at limit of detection, out of 18 samples per diet

^4^Data were not log-transformed. Presented are means and SED.
